# Prevalence of online sexual harassment and online bullying: a nationwide survey among high school students in Denmark

**DOI:** 10.3389/fpubh.2024.1368360

**Published:** 2024-08-07

**Authors:** Maj Britt Dahl Nielsen, Veronica Pisinger, Amalie Oxholm Kusier, Janne Tolstrup

**Affiliations:** The National Research Institute of Public Health, University of Southern Denmark, Copenhagen, Denmark

**Keywords:** cyberbullying, online bullying, online sexual harassment, internet victimization, cyber violence, high school students, adolescents, young people

## Abstract

**Background:**

Using data from a nationwide survey among high school students in Denmark, the aim of the current study is to measure the prevalence of online bullying and online sexual harassment and assess gender and age differences in exposure.

**Methods:**

We used data from the Danish National Youth Study 2019, which is a nationwide web survey among high school students, including general, commercial, preparatory and technical high schools. Data were collected from January to April 2019 through a self-administered questionnaire in the classroom. A total of 29,086 students participated (response rate: 66%). The survey included questions about online sexual harassment (victimization and perpetration) and online bullying.

**Results:**

Approximately 11% of students reported receiving sexually offensive inquiries online, and about 10% received a sexual image/video of others without the subjects’ consent. Additionally, 4% experienced that other people shared a sexual image/video of them without their consent within the last 12 months. The most common type of online bullying was feeling ignored by others online (25%), followed by someone spreading rumors or writing nasty things about them (13%), and receiving threats/unpleasant messages (12%). Gender results were mixed.

## 1 Introduction

A large part of young people’s lives take place on online platforms. Social media constitute an essential element in young people’s social lives that helps confirm and support friendships and dating relationships (1, 2). The internet, however, has also introduced new ways of victimization, such as online sexual harassment and online bullying, both of which can have severe negative consequences for young people’s mental health, and research has linked online sexual harassment and online bullying with lower life satisfaction, depression, anxiety, aggression and suicidality, and self-harm (3–5). Thus, young people’s online activities have sparked concerns among teachers, decision-makers, and parents about online safety (6).

Online sexual harassment and online bullying have in common that both involve negative behaviors harming the target. Research indicates that that online bullying is a precursor to sexual violence and that online sexual harassment and online bullying share the same protective and risk factors (7). Given that online sexual harassment and online bullying are relatively new phenomena, there are still definitional inconsistencies throughout the literature. Researchers often use different terms to describe online bullying and online sexual harassment, such as cyberbullying, internet bullying, online sexual victimization, and online solicitation (8, 9). More specifically, online solicitation refers to the process of encouraging an online contact to discuss sex, share personal sexual information, or engage in sexual behavior online (10).

Sexual harassment is a broad concept that encompasses blatant sexual abuse and more subtle behaviors (11). Angela et al. (8), defines online sexual harassment as: “*any interpersonal interaction involving sexually explicit content that is sent or forwarded using digital technology and is perceived as unwanted by the victim.”* This definition highlights the sexual nature of behaviors, but other scholars emphasize the gendered nature of sexual harassment. Inspired by the Tripartite Model of sexual harassment, Scarduzio et al. (12) defined online sexual harassment as consisting of three distinct but interrelated phenomena: (1) gender-related behavior, such as unwelcome verbal comments and/or images that are specifically related to gender, (2) unwanted sexual attention, and (3) sexual coercion that include threats to harm or cyberstalking (11).

Online bullying also involves harmful behavior based on information and communication technologies, e.g., harassing messages, posting disparaging comments on a social networking site, posting humiliating images, or threatening/intimidating someone electronically (9). In some definitions, online bullying is restricted to willful and repeated harm, but other definitions do not include criteria of repetition and/or intent (9).

Some scholars argue that online victimization is merely an extension of offline or face-to-face harassment and bullying (8). This argument has been supported by research showing that there is often an overlap between online and offline victimization and perpetration. However, studies on polyvictimization remains scare, and research show that the relationship is complex (13, 14). Furthermore, some behaviors only exist online (e.g., forwarding sexual messages to third parties), and other behaviors can only exist offline, such as physical contact (8). Online behaviors also differ from face-to-face behaviors in at least three important aspects. First, perpetrators can remain anonymous online, which often results in much harsher language. Second, the internet amplifies the dissemination of information and images, which can easily be distributed to a mass audience, harming the victim’s public image, and hereby representing double victimization. Third, new and constantly evolving technologies also create new venues and possibilities for aggressive and harassing behaviors (6, 8, 9, 11).

Prevalence estimates of both online sexual harassment and online bullying vary considerably between studies (15), and estimates depend on the definition, the measurement method, and recall period (16). Research also points to considerable cross-cultural differences, typically with higher prevalence’s in the Nordic countries (11, 17, 18). These differences may reflect differences in access to the online world, but may also reflect differences in tolerance of negative online behaviors (11). Although, it is difficult to compare prevalence estimates, previous research shows that online victimization is common among young people. A meta-analysis from 2018 found that one in five young people aged 12.0–16.5 years, experience unwanted online exposure to sexually explicit material, and that one in nine experience online sexual solicitation (15). Moreover, a review based on representative population samples among member countries of the European Union found that online bullying rates range between 2.8–31.5% among young people aged 12–18 years (18), and globally, the prevalence range is between 14 and 58% among students under 18 years (19).

Previous research has shown that bullying and sexual harassment perpetration and victimization differ by gender and age. Smith et al. (20) found that boys are more often perpetrators of bullying, but that the male predominance decreases between 11 and 13 years and increases between 13 and 15 years. These fluctuations may be related to the onset of puberty and increased propensity to risk-taking and antisocial behaviors. Gender differences in bullying perpetration is seen for both online and offline bullying (21). While boys and girls have an equal risk of bullying victimization (20), girls are more often exposed to sexual harassment compared to boys (22). However, these results may not be generalizable to the online environment, and large cross-national variations have been observed in gender differences in bullying (21).

Online behaviors are constantly modified as technologies advance, and the list of aggressive behaviors therefore requires constant revision. In addition, protective adaptations are also evolving, such as educational interventions, legislation, and online control and security options (6). While rates of online sexual harassment seem to be decreasing, the opposite pattern is seen for online bullying (18, 19, 23). Thus, it is crucial to continuously monitor trends in children’s and young people’s digital activities and safety (6, 8, 9, 11).

Using data from a nationwide survey among high school students in Denmark, the aim of the current study is to measure the prevalence of online bullying and online sexual harassment and assess gender and age differences in exposure.

## 2 Materials and methods

We used data from the Danish National Youth Study 2019, a nationwide web survey among high school students in Denmark. All high school education is preparatory and qualifying for higher education in Denmark, including general, commercial, preparatory, and technical high schools (24). The Danish National Youth Study 2019 is an independent follow-up to the Danish National Youth Study 2014 (25). The survey monitors key aspects of students’ health and wellbeing, including physical and mental health; alcohol, tobacco, and cannabis use; sleep, diet, sexual behavior, social relationships, school life, and digital media use (24, 25).

In brief, data was collected from January to April 2019 through a self-administered questionnaire in the classroom at 50 general high schools, 32 preparatory high schools, 15 commercial high schools and 19 technical high schools. A total of 29,086 students participated in the study (20,287 general high school students, 2,113 preparatory higher school students, 4,027 commercial high school students and 2,659 technical high school students), corresponding to 66% of the students in the 88 participating schools (31% of invited schools). High school students in Denmark are typically between 16 and 19 years old, with a mean age of 17.8 years, while students at preparatory high school are typically older. About 44% of respondents were boys and 56% girls, and 56% were 18 years or older. Finally, 91% were Danes, 2.3% were immigrants and 6.5% were descendants (data not shown) (24).

The design and data collection procedures are described in detail elsewhere, including non-response analyses (24).

### 2.1 Measures

The Danish National Youth Study 2019 includes four items about online sexual harassment and six questions about online bullying, as shown in Table 1. The questions about online sexual harassment include verbal and graphical harassment, and the questions about online bullying cover graphical and verbal abuse, exclusion from online forums, and experiencing that someone has created a fake profile impersonating oneself.

**Table 1:** Overview over questions about harassment in the Danish National Youth Study 2019.

Item	Question	Possible answers
**Online sexual harassment**		
Received sexually offensive inquiries	Within the last 12 months, have you received sexually offensive inquiries on the internet or social media?	“No”, “Yes”, “Do not want to answer”
Shared a sexual image/video of others	Within the last 12 months, have you shared a sexual image/video of others, e.g., in their underwear or naked, without their consent?	“No”, “Yes”, “Do not know”
Others shared a sexual image/video of you	Within the last 12 months, have others shared a sexual image/video of you, e.g., in your underwear or naked, without your consent?	“No”, “Yes”, “Do not know”
Received a sexual image image/video of others	Within the last 12 months, have you have received a sexual image image/video of others, e.g., in their underwear or naked, without the person’s consent?	“No”, “Yes”, “Do not know”
Online bullying		
Received threats or unpleasant messages	Within the last 12 months, have you received threats or unpleasant messages?	“No”, “Yes, once”, “Yes, several times”
Someone spread rumors or wrote nasty things about you	Within the last 12 months, has someone spread rumors about you or wrote nasty things about you?	“No”, “Yes, once”, “Yes, several times”
Others posted a image of you against your will	Within the last 12 months, have others posted a image of you online against your will?	“No”, “Yes, once”, “Yes, several times”
Felt ignored by others	Within the last 12 months, have you felt ignored by others online?	“No”, “Yes, once”, “Yes, several times”
Others created a fake profile impersonating them	Within the last 12 months, has someone created a fake profile impersonating you??	“No”, “Yes, once”, “Yes, several times”
Others shared a message, image or an update on social media that made you feel exposed and bullied	Within the last 12 months, have others shared a message, image or an update on social media that made you feel exposed and bullied?	“Never”, “Seldom”, “Sometimes”, “Often”, “Very often”

### 2.2 Analyses

We used Pearson’s χ^2^-test to test differences between genders and students younger and older than 18 years of age, which is the age of majority in Denmark. All analyses were performed in STATA 17. Missing data was handled by listwise deletion.

### 2.3 Ethics statement

Participation in the survey was voluntary, which was informed to the invitees. In Denmark, register and questionnaire studies do not require approval by committees on biomedical research ethics according to Danish legislation. The survey in 2019 was approved by SDU Research & Innovation Organization (RIO). Since 2016, RIO examines and approves all scientific and statistical projects at the University of Southern Denmark according to the Danish Data Protection Regulation.

## 3 Results

### 3.1 Online sexual harassment

Table 2 shows the percentage of students who have been exposed to or engaged in (perpetration) of online sexual harassment. A total of 10.5% of the students reported having received sexually offensive inquiries online within the last 12 months (Table 2). The proportion was higher for girls (14.8%) than boys (4.9%). Moreover, 9.7% reported having received an image or video of a sexual nature (henceforth: sexual image/video) of others without the subjects’ consent and 4% reported that others had shared a sexual image/video of them without their consent. The proportion of boys who experienced that other people shared a sexual image/video of them (4.6%) without their consent and who received a sexual image/video of others without the subjects’ consent (11.8%) was statistically significantly higher than among girls.

**Table 2:** Prevalence of students who have experienced or engaged in online sexual harassment within the last 12 months in total and divided by gender.

Online sexual harassment	Total population					Boys					Girls					
	N	Yes	%	Do not want to answer	%	N	Yes	%	Do not want to answer	%	N	Yes	%	Do not want to answer	%	*p* ^*^
Received sexually offensive inquiries	26,843	2,807	10.5	501	1.9	11,743	572	4.9	262	2.2	15.100	2.235	14.8	239	1.56	0.000
	N	Yes	%	Do not know	%	N	Yes	%	Do not know	%	N	Yes	%	Do not know	%	
Shared a sexual image/video of others	26,801	471	1.8	543	2.0	11,720	340	2.9	377	3.2	15,081	131	0.87	166	1.1	0.000
Others shared a sexual image/video of you	26,795	1,071	4.0	630	2.4	11,715	539	4.6	408	3.5	15,080	532	3.5	222	1.5	0.000
Received a sexual image image/video of others	26,788	2,593	9.7	748	2.8	11,705	1,381	11.8	490	4.2	15,083	1.212	8.0	258	1.7	0.000

*p-value for χ^2^ test.

Only a few respondents (1.8%) reported having shared a sexual image/video of others without the subjects’ consent. Among boys, 2.9% reported having shared a sexual image/video of others without the subjects’ consent, and 3.2% did not know whether they had shared a sexual image/video of others without the subjects’ consent. The proportion was lower among girls. There were no major differences in the experience of online sexual harassment across students younger and older than 18 years of age (Table 3).

**Table 3:** Prevalence of students who have experienced or engaged in online sexual harassment within the last 12 months, divided by age.

Online sexual harassment	<18 years					≥18 years					
	N	Yes	%	Do not want to answer	%	N	Yes	%	Do not want to answer	%	*p* ^*^
Received sexually offensive enquiries	11,831	1,271	10.7	205	1.7	15,012	1,536	10.2	296	2.0	0.155
	N	Yes	%	Do not know	%	N	Yes	%	Do not know	%	
Shared a sexual image/video of others	11,806	221	1.9	218	1.9	14,995	250	1.7	325	2.2	0.085
Others shared a sexual image/video of you	11,805	491	4.2	263	2.2	15,890	580	3.9	367	2.5	0.252
Received a sexual image image/video of others	11,802	1,223	10.4	320	2.7	14,986	1,370	9.1	428	2.9	0.003

*p-value for χ^2^ test.

### 3.2 Online bullying

Tables 4, 5 show the prevalence of students experiencing online bullying (by gender and age). A total of 9.2% of the students reported having experienced someone spreading rumors or writing nasty things about them once, and 4% more than once within the last 12 months. For most questions gender differences were found, with some items being more prevalent among boys and others more prevalent among girls. For example, more boys reported that they received threats and unpleasant messages, whereas more girls reported feeling ignored by others online. Compared to students aged 18 years and above (Table 5), students younger than 18 years old more frequently reported receiving threats or unpleasant messages; that someone spread rumors or wrote nasty things about them; that others posted an image of them online against their will; and that others created a fake profile impersonating them.

**Table 4:** Prevalence of students who have experienced online bullying within the last 12 months in total and divided by gender.

Online bullying	Total population					Boys					Girls					
	N	Yes, once	%	Yes, more than onetimes	%	N	Yes, once	%	Yes, more than once	%	N	Yes, once	%	Yes, more than once	%	*p* ^*^
Received threats or unpleasant messages	26.274	2,111	8.0	938	3.6	11,416	1,038	9.1	572	5.0	14,858	1,073	7.2	366	2.5	0.000
Someone spread rumors or wrote nasty things about you	26.257	2,424	9.2	1,044	4.0	11,408	907	8.0	396	3.5	14,849	1,517	10.2	648	4.4	0.000
Others posted a image of you online against your will	26.237	1,748	6.7	573	2.2	11,399	704	6.2	261	2.3	14,838	1,044	7.0	312	2.1	0.014
Felt ignored by others online	25.847	4,168	16.1	2,347	9.1	11,147	1,376	12.3	772	6.9	14,700	2,792	19.0	1,575	10.7	0.000
Others created a fake profile in your name	26.223	955	3.6	273	1.0	11,387	436	3.8	123	1.1	14,836	519	3.5	150	1.0	0.311
	N	Sometimes/Seldom	%	Very often/often	%	N	Sometime/Seldom	%	Very often/often	%	N	Sometimes/Seldom	%	Very often/often	%	
Others shared a message, image, or an update on social media that made you feel exposed and bullied	25,839	4,680	18.1	218	0.84	11,149	2,054	18.4	137	1.23	14,690	2,626	17.9	81	0.55	0.000

*p-value for χ^2^-test.

**Table 5:** Prevalence of students who have experienced online bullying within the last 12 months, divided by age.

Online bullying	<18 years					≥18 years					
	N	Yes, once	%	Yes, more than once	%	N	Yes, once	%	Yes, more than once	%	*p* ^*^
Received threats or unpleasant messages	11,584	983	8.5	466	4.0	14,690	1,128	7.7	472	3.2	0.000
Someone spread rumors or wrote nasty things about you	11,580	1,182	10.2	512	4.4	14,677	1,242	8.5	532	3.6	0.000
Others posted a image of you online against your will	11,567	884	7.6	294	2.5	14,670	864	5.9	279	1.9	0.000
Felt ignored by others online	11,378	1,892	16.6	1,038	9.1	14,469	2,276	15.7	1,309	9.1	0.133
Others created a fake profile in your name	11,563	470	4.1	126	1.1	14,690	485	3.3	147	1.0	0.004
	N	Sometimes/ Seldom	%	Very often/often	%	N	Sometime/ Seldom	%	Very often/ often	%	
Others shared a message, image or an update on social media that made you feel exposed and bullied	11.386	1,987	17.5	98	0.86	14,453	2,693	18.6	120	0.83	0.049

*p-value for χ^2^-test.

## 4 Discussion

This study contributes with knowledge about online victimization and perpetration based on data from a nationwide study among high school students in Denmark. We find that online sexual harassment is relatively common among students, with one in five students having received sexually offensive inquiries online. This phenomenon is also known as online sexual solicitation (10). While online sexual solicitation could be initiated by peers, it is important to note that it is also a common method used to manipulate minors to engaging in sexual relationships (10).

Our estimate of the prevalence of online solicitation closely aligns with the meta-analysis by Madigan et al. (15) on online solicitation among young people aged 12–16.5 years. Like Madigan et al. (15), we find no age differences in the prevalence. Although older adolescents may spend more unsupervised time online and thus be more likely to be exposed, they may also be less inclined to perceive the contact as unwanted or unsolicited (23). In contrast to our study, Madigan et al. found that online solicitation was higher among boys. However, our findings are comparable to a previous study among Danish school children aged 14–17. That study reported that approximately 16% of girls and 5% of boys experienced online solicitation by an unknown person during the last year (26).

The current study also sheds light on practices related to sharing nude images/videos. While these practices are often framed as normal behavior that allows youths to explore sexual identity and initiate new affective or sexual relationships, they also entail the risk of non-consensual distribution of sexual images/videos (27).

In Denmark, sharing nude images/pictures without the consent of the subject is illegal (and sharing of nude images of people under 15 years old is illegal in any case). We find that about 2% of students in our study reported sending an image/video without the subjects’ consent, which is much lower than findings from previous meta-analyses where prevalence’s ranged between 12 and 15% (15, 23, 28–30). We also found a lower prevalence of students who experienced other sharing a sexual image/video of them without consent (4%) compared to previous research. Previous meta-analyses have found a prevalence of having a sext forwarded without consent to be about 8 to 9% (15, 23, 28–30). The reason for these differences, might be related to the unfolding of the “Umbrella case” in Denmark in 2018 when the police charged more than 1,000 people for sharing nude images of a 15-year-old girl. As this case received substantial national media coverage, it likely affected young people’s perceptions and practices regarding sharing nude images, which may be partially reflected in the results of our study, as the data were collected during the case’s development. Interestingly, the percentage reporting not knowing if they had shared a sexual image/video of others without the subjects’ consent was slightly higher than the proportion who reported sharing such content. This discrepancy might reflect social desirability bias but could also indicate a lack of knowledge about the law.

Like previous studies we do not find significant age differences in the prevalence of nonconsensual sharing of nude images/videos (except of receiving sexual images/video without the subjects consent, which was slightly higher among students under 18 years), but in contrast to previous studies, we find that boys are more likely to be both victims and perpetrators of nonconsensual sharing of nude images/videos (15, 23, 28–30).

Previous research on face-to-face sexual harassment has rather consistently shown that girls and women are more likely to be exposed to sexual harassment—e.g., by strangers in the streets and public spaces, by colleagues and managers at work, and by peers and professors at the universities (31–33). Thus, an obvious question is why our study shows a somewhat different pattern regarding online sexual harassment. One explanation may be related to differences in boys’ and girls’ online behavior (34, 35). In our study, we found that boys were more likely to engage in online sexual harassment perpetration, which could be perceived as a type of online sexual risk behavior. Another explanation may be that current surveys on online sexual harassment primarily focus on behaviors of a more sexual nature rather than gender-based harassment. We found that girls are more likely to experience someone spreading rumors or writing nasty things about them. It is likely that these nasty messages include gender-based harassment. Finally, girls may be more reluctant than boys to report online sexual harassment, because they may be more inclined to self-stigmatization and self-blaming (36). Previous research has demonstrated how gendered norms and taboos play a significant role in non-consensual image sharing. Girls’ images are more likely to be evaluated as sexual, and girls are more likely to be judged and shamed than boys. This double standard underpins the victim-blaming of girls, who are often made responsible for the misuse of their images, while boys can gain respect through the possession of images. These images have a high value on the digital marketplace, and contributes to boys’ popularity (37). Similarly, a previous qualitative study among young people in Denmark showed that non-consensual sharing acts as a form of visual gossip to maintain social bonds and gendered recognition (38).

In the current study, we lack information about the perpetrator. Consequently, we do not know whether online harassment was conducted by a stranger or a familiar person, nor whether it is merely an extension of face-to-face harassment. Previously, Helweg-Larsen, Schütt, and Larsen (26) found that relatively few children had experienced offline harassment from people they knew through the internet. In contrast, a large European study on sexual harassment among women aged 18–24 found that online harassment is inflicted most often by those who are the best known (11).

Previous research on online bullying has produced prevalence’s rates that differs considerably. For instance, Zhu et al. (19) found that online bullying victimization ranges between 14 and 60% globally, while Henares-Montiel et al. (18) reported ranging between 3 and 32% in the EU. The most common type of online bullying was feeling ignored by others online (25%), followed by someone spreading rumors or writing nasty things about them (13%), and receiving threats/unpleasant messages (12%) at least one time during the last 12 months. The experience of having rumors spread online was also observed in a study among Danish adolescents (13–17 years old), where the prevalence of 12%, closely align with our findings (26).

Our study has also contributed with knowledge about age and gender differences in online bullying. Globally, girls are more likely to be exposed to online bullying (19), but research on gender differences in a European context remains scarce (18). In our study. we found that boys more often reported receiving threats or unpleasant messages, while girls more often reported feeling ignored by others. We found no gender differences regarding having a fake profile set up. In general, young people under 18 were more likely to experience online bullying.

It is a strength that we used data from a nationwide study. The Danish National Youth Study 2019 provides unique data about high school students’ physical and mental health and detailed data about online sexual harassment and online bullying. Thus, our study provides researchers and practitioners with a unique opportunity to use data for planning health education and health promotion interventions. However, the study also has some shortcomings. Most importantly, the measurement of online sexual harassment and online bullying was not based on a standardized instrument but was developed for the study. Furthermore, because the survey was designed to measure a range of topics, it does not include all relevant aspects of the online sexual harassment and bullying. First, the questions about online sexual harassment only included questions about sexual behaviors and not gender-based harassment. In traditional sexual harassment research, gender-based harassment is generally considered a crucial element in sexual harassment, and previous studies have consistently shown that gender-based harassment is the most common type of sexual harassment (33). Second, we only had question on online perpetration; thus, this study provides very little information on such behaviors. Third, our study did not include questions about the identity of the perpetrator. Although the victim may not always know who the perpetrator is, we agree with Angela et al. (8) that future surveys should include questions about the identity and gender of the perpetrator to establish whether the harassment and bullying is perpetrated by adults or peers or boys or girls. This knowledge is important for designing effective preventive efforts.

Although the survey includes separate questions about online sexual harassment and online bullying, there might be an overlap between the questions, e.g., questions about others posting images of them without consent. Further, it is debatable if a single negative experience constitutes bullying and not just a negative experience. Repetitiveness, intentionality, and power imbalance are commonly accepted definitional characteristics of traditional (offline or face-to-face) bullying. In online bullying, receptiveness takes on a different meaning, as a single post, message or video can go viral. Moreover, defining power imbalances in the online context is often difficult because the bully can remain anonymous online. Finally, intentionality is generally difficult to establish (even in traditional bullying), because practices are open to cover-ups and misunderstandings (14).

The questions in our study vary considerably regarding the level of seriousness, e.g., receiving threats or feeling ignored. However, we are unable to conclude whether some experiences are more detrimental than others, or if a single episode can have a negative impact on mental health. More research on the impact on mental health could be helpful to assess whether the frequency of exposure mediates the negative impact and if certain behaviors are more detrimental than others. Finally, this study is based on data from 2019. Considering the rapid changes in the online environment, the data might already be considered a bit dated.

To date, preventive interventions directed at young people primarily fall into two categories: online safety education and sex and relationship education. A review from 2011 found that interventions that focus on raising awareness about internet safety can increase students’ knowledge, but not change their attitudes and behaviors (39). Although sex and relationship education traditionally focuses on prevention of sexually transmitted diseases (40), research shows that students’ sexual harassment behaviors can be reduced through dedicated school lessons (39, 41). Our findings suggest that young people need more knowledge about the sharing of images; also because a substantial proportion was unsure whether they had shared images of others against their will.

Supporters of sex and relationship education argue that online sexual harassment should be seen as an extension of offline sexual harassment, as most victims know their harasser from the physical world. Moreover, some studies show that polyvictimization (being exposed to different types of victimization in multiple settings, e.g., the home, school, and community) is common. This could call for broader psychosocial interventions that target a wider range of negative behaviors (41). To design effective interventions, we therefore need more research about the characteristics of online sexual harassment, i.e., risk factors and polyvictimization, and more knowledge about the effectiveness of preventive interventions. This knowledge, however, should be combined with an in-depth understanding of the needs and challenges of young people and their parents and teachers to ensure that preventive interventions are tailored to their needs and that the implementation will be feasible in practice.

## Data availability statement

The raw data supporting the conclusions of this article will be made available by the authors, without undue reservation.

## Ethics statement

Ethical approval was not required for the studies involving humans because in Denmark, register and questionnaire studies do not require approval by committees on biomedical research ethics according to Danish legislation. The survey in 2019 was approved by SDU Research & Innovation Organization (RIO). Since 2016, RIO examines and approves all scientific and statistical projects at the University of Southern Denmark according to the Danish Data Protection Regulation. The studies were conducted in accordance with the local legislation and institutional requirements. Written informed consent for participation was not required from the participants or the participants’ legal guardians/next of kin in accordance with the national legislation and institutional requirements because the study is based on survey data. Written consent from guardian is not required by the Danish law to collect survey data. Participation in the study was voluntary.

## Author contributions

MN: Conceptualization, Writing – original draft. VP: Data curation, Formal analysis, Writing – original draft. AK: Conceptualization, Writing – original draft. JT: Conceptualization, Project administration, Writing – review & editing.
